# Dual Functional Capability of Dendritic Cells – Cytokine-Induced Killer Cells in Improving Side Effects of Colorectal Cancer Therapy

**DOI:** 10.3389/fphar.2017.00126

**Published:** 2017-03-14

**Authors:** Paula Mosińska, Agata Gabryelska, Malwina Zasada, Jakub Fichna

**Affiliations:** ^1^Department of Biochemistry, Faculty of Medicine, Medical University of ŁódźŁódź, Poland; ^2^Department of Cosmetic Raw Materials Chemistry, Faculty of Pharmacy, Medical University of ŁódźŁódź, Poland

**Keywords:** dendritic cells, cytokine-induced killer cells, colorectal cancer, immunotherapy, colorectal cancer treatment

## Abstract

The aim of cancer therapy is to eradicate cancer without affecting healthy tissues. Current options available for treating colorectal cancer (CRC), including surgery, chemotherapy or radiotherapy, usually elicit multiple adverse effects and frequently fail to completely remove the tumor cells. Thus, there is a constant need for seeking cancer cell-specific therapeutics to improve the course of cancer therapy and reduce the risk of relapse. In this review we elaborate on the mechanisms underlying the immunotherapy with dendritic cells (DCs) and cytokine-induced killer (CIK) cells, and summarize their effectiveness and tolerability available clinical studies. Finally, we discuss the up-to-date combinatorial adoptive anti-cancer immunotherapy with CIK cells co-cultured with DCs that recently showed encouraging efficacy and usefulness in treating malignant disease, including CRC.

## Introduction

Colorectal cancer is the second in woman and the third in men most commonly diagnosed cancer worldwide (Siegel et al., 2015), accounted for approximately 1.2 million new cases and over 600.000 deaths annually (Torre et al., 2015). Surgical resection is the first choice procedure for patients suffering from CRC, and is frequently followed by radio- or chemotherapy. For early staged CRC patients, the combined course of treatment results in 5-year survival rate of up to 80% (Zhu et al., 2014). Nevertheless, surgical resection with adjuvant radio- or chemotherapy is often poorly tolerated by patients due to many severe side effects directly related to complications after surgery, including infections and bleedings, and indirectly to psychological trauma, nausea, vomiting, and fever. Therefore, new, more patient friendly and especially effective treatment is necessary to avoid tumor recurrence, and enhance the response rate to therapy.

Colorectal cancer is a heterogenous disease in terms of its molecular characteristics, clinical manifestations, and sensitivity to treatments. Along with accumulation of genetic alterations (e.g., genomic and chromosomal instabilities) and epigenetic changes that constitute the main forces for tumor development, immune pattern of the tumor microenvironment seems to be the major predictor of patient’s survival in a variety of primary tumors (Maby et al., 2015). High density of T cells and CD8^+^ T cells cytotoxic orientation or mature DCs are considered as a target in the treatment of large group of cancers such as colorectal, lung, breast, pancreatic and melanoma cancers (Al-Shibli et al., 2008; Pagès et al., 2009; Mahmoud et al., 2011; Remark et al., 2013). Adaptive and innate immune system can protect the host from tumor development through mechanisms of immunosurveillance (Mlecnik et al., 2010; Stoll et al., 2015). Cancer immunotherapy involves the use of therapeutic modalities, which stimulate the host’s anti-tumor response by altering the effector cell number and secretion of soluble mediators, and decreases the host’s suppressor mechanisms by modulating immune checkpoints. The immunotherapy represents the most promising new cancer treatment approach, also among CRC patients. Patients suffering from CRC are in an immunosuppressed state when they undergo radiotherapy, surgery, or chemotherapy and often exhibit compromised immune responses; therefore, it is recommended to target dysfunctional cells to achieve most promising therapeutic results and simultaneously recover anti-cancer immunity to support immune response to tumor cells. DCs, responsible for the activation of T-cells, and CIK cells, which induce the secretion of a diverse array of cytokines, are rapidly evolving immunotherapeutic targets in eradicating residual cancer cells (Yang et al., 2011). The therapy has already been used in the treatment of chronic myeloid leukemia and other types of cancer such as liver, kidney, breast, and prostate (Wang et al., 2009).

Understanding of the molecular interactions between immune system and tumor cells has significantly improved in the last decade, leading to the development of DC-CIK adoptive immunotherapy, which encompasses the introduction of DCs in combination with the CIK cells. The immunotherapy with DC-CIK reduces the severity of adverse effects, namely grade III and IV leukopenia, thrombocytopenia and anemia, which commonly occurs as a consequence of conventional cancer therapy, improves overall quality of life and prolongs the survival of CRC patients (Lin et al., 2016).

## Mechanism of Action of DCs

Dendritic cells are well-characterized APCs (Steinman, 1991), which reside in peripheral tissues. The role of DCs is to capture, process and present phagocytosed antigens, including TAAs, express lymphocyte costimulatory molecules and secrete cytokines, such as IL-12, IL-15 and type I interferons (IFNs I) to initiate primary immune response in resting naive T cells (Pulendran et al., 2008; Legitimo et al., 2014).

**FIGURE 1:**
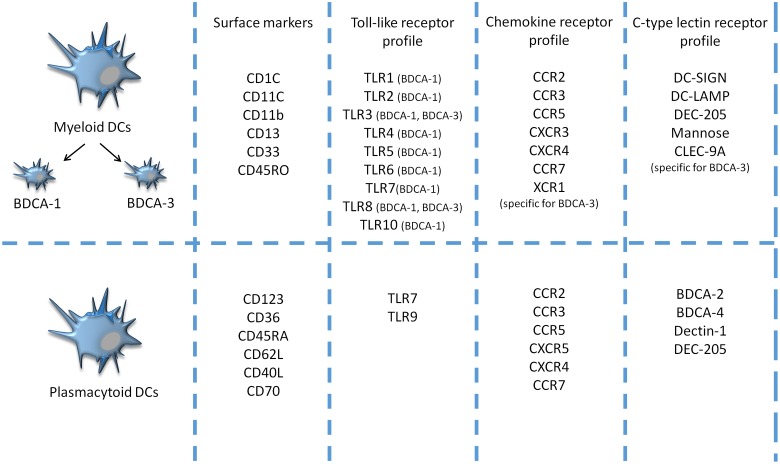
**Comparison of surface markers, toll-like receptor (TLR), chemokine receptors (CCR) and C-type lectin receptors (CLRs) profiles between two major populations of naturally circulating DCs- human blood myeloid and plasmacytoid DCs- in the peripheral blood of humans (Mckenna et al., 2005; Bamboat et al., 2009; Reizis et al., 2011)**.

Dendritic cells comprise multiple subsets with different morphologic, phenotypic, and functional properties. There are two main classes of DCs, similar for both mice and humans: mDCs, also called classical DCs, and pDCs, which originate from CD34+ bone marrow precursors (**Figure 1**). Under certain conditions, both types can be also propagated from monocytes (Shortman and Naik, 2007; Pulendran et al., 2008). The two DC subsets possess distinct features: mDCs express CD11c marker and are considered as principal producers of IFNs I, whereas pDCs express mainly CD123 and IDO, an enzyme participating in the generation of regulatory T cells (Koido et al., 2013). A detailed discussion of DC subtypes can be found elsewhere (Dudek et al., 2013; Ma et al., 2013; Legitimo et al., 2014). In healthy conditions, DCs occur in an immature or semi-mature state and are usually localized in various non-lymphoid organs and tissues. Immature DCs are responsible for the uptake and process of peptides. They are able to respond to various inflammatory signals, e.g., toll-like receptors (TLR), NOD-like receptors, scavenger receptors, and inflammatory mediators, chemokines and cytokines (Niu et al., 2014). Upon activation, immature DCs migrate to lymphoid tissues and interact with T cells. Active DCs produce and secrete IL-12, which induces cell differentiation of lymphocytes T CD4 + into antigen-specific effector Th cells e.g., Th1, Th2, Th17, and increases production of interferon-gamma (IFN-γ) and cytotoxic activity of CTL, essential for antitumor immune activity (Koido et al., 2013). The distinction between mature and immature DCs relates to their phenotypic and functional properties. Maturation of DCs occurs when the cell can upregulate MHC class II molecules and several surface ligands, including CD80, CD83 and CD86 (Märten et al., 2001). Moreover, fully matured DCs can control the balance between inflammatory, e.g., IL-6, immunostimulatory, e.g., IL-12 cytokines and immunosuppressive cytokines (Xie et al., 2006). However, existing evidence suggests that DCs may occur in states, since they exhibit high functional plasticity, i.e., DCs are able to express immunostimulating and/or immunesuppresive factors depending on microenvironmental conditions that affect their differentiation, maturation, polarization and activation; however, even in multiple environmental milieus morphologically different DC subsets are able to stimulate T cell proliferation and modulate their response (Manicassamy and Pulendran, 2011).

Various endogenous and exogenous stimuli affect maturation of DCs by changing the processing and presentation of the antigen, e.g., activated natural killer (NK) cells acquire the ability to defeat DCs that have failed to undergo complete maturation. Moreover, DC–NK interaction induces generation of various cytokines by both cell types, and besides enhancing DC maturation, also promotes the proliferation of NK cells. Endogenous antigens are degraded into peptides by proteasomes in the cytosol and together with TAP are transferred to ER, where they create complexes with MHC class I molecules. The peptide-MHC I complexes is subsequently presented to CD8+ T cells. This form of antigen presentation is available not only for DCs but also for other nucleated cells (Ma et al., 2013). Exogenous antigens in turn are processed in endosomes, loaded onto MHC class II molecules, and activate CD4+ T cells and cause their further polarization to Th cells. This process is restricted only to APCs (Ma et al., 2013).

Metastasis and tumor stage are related to higher infiltrations of DCs in the tumor tissue (Gordon et al., 2014). DCs modulate innate and adoptive immunity, affect oncogenesis, especially tumor progression, and therefore influence response to cancer therapy (Bloy et al., 2014). However, the presence of tumor cells may also drive DCs into state of tolerance and immunosuppression, resulting in their decreased or completely inhibited activity of initiating the immune response. Functional impairments of DCs have been reported in various types of cancer, such as chronic myeloid leukemia, liver cancer as well as CRC (Miller et al., 2004).

Dendritic cell-based interventions aim at (re)activating the endogenous cancer-specific immune response, which *de facto* constitutes an anti-cancer vaccine. Exploiting naturally circulating DCs can be performed either by isolating pDCs or mDCs and stimulating them *ex vivo* (with adjuvants and antigens), *in vivo* (by means of nanoparticles coated with antibodies against DCs-specific cell surface receptors), and DC-derived exosomes (Bol et al., 2013; Bloy et al., 2014).

## General Mechanism of CIK Cells

Cytokine-induced killer cells are *ex vivo* expanded heterogeneous population of lymphocytes T CD8+ with additional NK cells phenotype. They are generated by an *in vitro* extraction of PBMC and its further exposition to IFN-γ, anti CD-3 antibody, and prolong propagation in presence of high-dose IL-1 and IL-2 (Wang et al., 2011; Schlimper et al., 2012). After maturation, when the majority of cells exhibit granular lymphocyte morphology and express NK and T-cell markers, CIK cells are transferred to the recipient in autologous or allogeneic settings (Zhao et al., 2015).

The lytic activity of CIK cells is driven mainly by CD3^+^CD56^+^ cells that can coexpress both the T-cell marker CD3 and the NK cell marker CD56 (Pievani et al., 2011). They were first described in 1991 by Schmidt-Wolf et al. (1991), who concluded CIK cells to have higher proliferation and strengthened cytotoxic properties against tumor cells compared to their progenitors LAKs. CIK cells display non-MHC-restricted NK-like anti-tumor activity against allogenic and syngeneic hematological malignancies (Sangiolo et al., 2008). They secrete proinflammatory cytokines, predominantly IFN-γ and IL-4. Due to their CLT phenotype, CIK cells are also capable of eliminating cells presenting MHC molecules (Wang et al., 2009). Mechanisms used by CIK cells to terminate cancer cell include release of granules, such as granzymes and perforin. Along with NK cells, macrophages, monocytes, and neutrophils, CIK cells can mediate cytotoxicity through ADCC (Kohrt et al., 2012). It is therefore possible that besides recognizing cancer cells without prior antigen sensitization in a non-MHC restricted manner, CIK cells anti-tumor activity might be enhanced by adding specific monoclonal antibodies targeting ADCC (Wang et al., 2014). Since CIK cells can be produced by a simple approach and exert anti-tumor activity *in vitro*, they seem to be suitable tools for the treatment of solid and hematopoietic tumors (Li et al., 2012). Autologous and allogenic CIK cells have been already evaluated in the treatment of different tumor types (NCT0076910, NCT01186809, NCT02886897, NCT00394381, NCT00815321, NCT01871480, and NCT01232062).

## DC–CIK Interaction

Based on available data the adoptive immunotherapy with DC-CIK encompass either the infusion of DCs (the amount of infused cells ranges from 1 to 5 × 10^7^) following the administration of CIK cells (the amount of cells range from 2 to 15 × 10^9^) or autologous tumor lysate pulsed DC-CIK cells infused intravenously (NCT02202928) (Zhu et al., 2014).

According to different clinical trials, DCs can be injected subcutaneously, intravenously or intradermally, in turn CIK cells are usually administered intravenously or intradermally (NCT01956630, NCT02491697, and NCT02202928).

**FIGURE 2:**
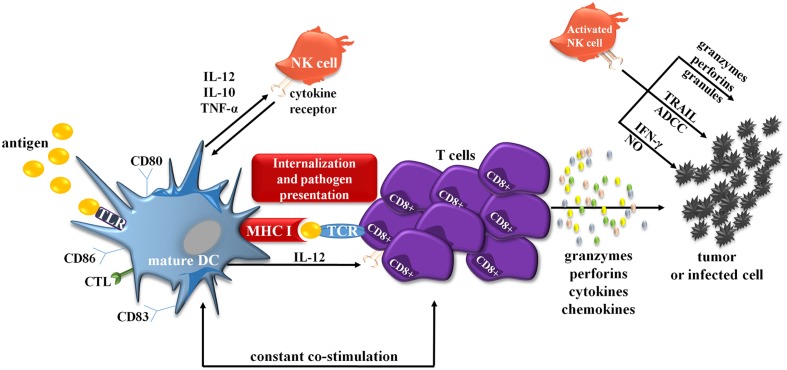
**Simplified scheme showing interactions between DCs and CIK cells. mDCs are also able to crosstalk with NK cells, prototypical effector cells of innate immunity.** Both mDC and NK cells reciprocally stimulate and regulate their functions. For example upon activation, NK cells acquire the ability to defeat immature DCs. On the other hand, activated IFN-α-generating DCs can also stimulate NK cells and initiate antigen-specific T- and B-cell responses. The crosstalk appears via cell-to-cell interaction and largely involves the activation of NK cells via transmembrane TNF-α. Activated NK cells can also directly kill target tumor cells through several mechanisms involving TRAIL, ADCC, IFN-γ, NO or by the release of granzymes, perforins, granules. ADCC, antibody-dependent cellular cytotoxicity; CTL, C-type lectins; IFN-γ, interferon γ; MHC II, major histocompatibility complex II; NK cell, natural killer cell; NO, nitric oxide; TCR, toll-like receptors; TRAIL, tumor necrosis factor-related apoptosis-inducing ligand.

Any dysfunction of either DC or CIK cells may cause inefficient immune response, which consequently enables cancer cells to spread. Interactions between molecules on the surface of mature DCs and CIK cells cause an increased activation of each of these populations; however, the exact mechanism of action is still not fully understood. The stage of DCs maturation determines DC–CIK interaction and its function (Lin et al., 2016). Mature DCs provide necessary signals for proliferation of CIK cells by increasing their cytotoxic and cytolytic capabilities; immature DCs are unable to trigger this reaction and are consequently killed by CIK cells (Qu et al., 2014). DC–CIK interaction also causes a significant increase in the number of costimulatory and antigen-presenting molecules on DCs surface, which provides the cell with greater tumor-fighting capabilities (**Figure 2**). Combination of DC-CIK in cancer therapy, compared to each therapy alone, results in an enhanced immune response in treated patients (*n* = 100) and greater therapeutic effects (Zhu et al., 2014). Numerous studies provide evidence that DC-CIK were effective in the treatment of multiple solid tumors, e.g., breast cancer, non-small-cell lung cancer, without causing any serious adverse reactions.

The fact that distinct subsets of DCs precursors can be activated and mobilized by various cytokines, e.g., in humans pDCs can be regulated *in vivo* with Flt4 ligand or G-CSF, whereas other blood DC subsets only by Flt3L, it additionally provides an individual approach for manipulating of immune responses in humans (Arpinati et al., 2000; Pulendran et al., 2000). This approach may constitute another way of improving the effect of therapy with DC-CIK.

However, it has to be mentioned that multiple parameters can modulate the quality of immune responses through DC targeting (Ueno et al., 2011). Thus, some major issues need consideration during DC vaccine development:

-biological function of the DC subsets that is anticipated to deliver antigens,

-the type of activation signaling and immune response that the DC can induce,

-the selection of antigens and their formulation that will induce the antigen-specific response

-the receptors expressed by the particular subset of DCs.

## Beneficial Effects of DC-CIK Therapy

One of great advantages of immunotherapy is an easy evaluation and monitoring of patients’ response to therapy based on DTH reaction. According to Zhu et al. (2014; Lin et al., 2016), among 100 patients with CRC, more than 60% developed positive cell-mediated cytotoxicity response to the treatment with DC-CIK, assessed in DTH skin test. Adverse events occurred in less than 30% of recruited patients and included fever, insomnia, sore joints and skin rush (Niu et al., 2014; Zhang et al., 2016). However, validity and efficacy of DTH can be affected by the tumor environment and therefore the obtained results should be treated with caution.

Dendritic cell-cytokine-induced killer cell-based immunotherapy combined with chemotherapy prolongs PFS in patients with CRC, and increases OS rate to almost 4 months, when compared to chemotherapy alone (Lin et al., 2016). Similar outcomes were reported in the study by Niu et al. (2014) in which 44 out of 70 patients with CRC developed positive immune response after DC-CIK-based immunotherapy, which prolonged their MST. No severe adverse events were observed; however, similarly to the study by Zhu et al. (2014) patients experienced mild inconveniences, e.g., insomnia, fever, anorexia, skin rush or joint soreness. The respective increase in PFS and OS were almost 9 and 15 months, respectively, for patients treated with DC-CIK therapy additionally to chemotherapy. The incorporation of the adjuvant immunotherapy for patients undergoing primary chemotherapy resulted in general improvement in quality of life, including physical strength, appetite, better sleep and increase in body weight (Zhan et al., 2012). Finally, DC-CIK therapy proved to decrease the recurrence and metastasis rate in CRC patients (Lin et al., 2016).

Dendritic cell-cytokine-induced killer therapy targets tumor cells without attacking other cells of the organism, which it is not associated with toxicity, and thus make it a viable treatment option for patients in poor health (Hui et al., 2009). As opposed to chemotherapy, DC-CIK therapy induces fewer and less severe adverse effects, and also limits the side effects caused by chemotherapy itself. Adjuvant DC-CIK therapy to primary chemotherapy lowers the frequency of severe treatment induced complications – hematological toxicities such as leukopenia, anemia and thrombocytopenia as well as nausea, vomiting and abdominal liver function (Lin et al., 2016).

Worth mentioning, tumors that develop radiation or chemotherapy resistance, still remain a suitable target for immunotherapy (Dasgupta et al., 2011). A combined approach to use conventional anti-cancer therapy to kill the bulk of cancer cells and immunotherapy (e.g., DCs as adjuvants) to improve immunization against tumor cells, can be even more effective in treating cancer.

Currently, there are few clinical trials, both ongoing and still recruiting, assessing the effect of the autologous tumor lysate pulsed DC-CIK treatment in CRC patients (NCT02202928, NCT01839539, and NCT02415699).

## Limitations of Anti-CRC Therapy

Chemotherapy has many imperfections: not only its sensitivity declines over time, but often causes numerous adverse effects, for example nausea and vomiting. This line of treatment is also associated with severe toxicity, which deteriorates general health of patients and leads to discontinuation of treatment (Dasgupta et al., 2011).

In contrast to chemotherapy, the side effects of immunotherapy include insomnia, fever, anorexia, diarrhea, joint soreness and general fatigue (Wood et al., 2000). They are minor, occur rarely and are mostly self-resolving; usually no special treatments and hospitalizations are necessary. In some cases, the anti-inflammatory drugs are used to resolve side effects such as fever. The treatment with DC-CIK does not prompt autoimmune reactions (Niu et al., 2014). In some singular cases limited toxicity may appear; however, lowering the dose of the treatment resolves the side effects and allows the patients to continue the treatment (Zhan et al., 2012). Nonetheless, because the positive cell-mediated cytotoxicity response to DC vaccine and CIK cell therapy is developed in around 60% of CRC patients, the therapy is not effective for all (Niu et al., 2014).

Currently, peripheral blood is the main source of DC-CIK. As the therapy should be personalized for each patient, the repeated collection of blood can be difficult and may imply a considerable costs, which narrow the number of recipients. In some cases, e.g., elderly patients or patients in poor health, obtaining the number of cytotoxic cells necessary for the therapy may pose a significant challenge and limit the utility of the therapy.

There is still insufficient number of clinical trials that evaluate the effectiveness of DC-CIK therapy in patients with CRC, and which determine the probability of the occurrence of adverse effects. Randomized controlled trials with larger sample sizes or meta-analysis are warranted to provide evidence for further application of this therapy.

## Conclusion

Immunotherapy has fewer side effects than standard treatment with chemo- or radiotherapy and is generally more potent in preventing their onset. Recent studies present new treatment options for patients suffering from CRC, focusing predominantly on DC-CIK therapy, which stimulate the immune system of a patient against cancer. Being in the spotlight of cancer prevention, the therapy with DC-CIK shows a great potential in alleviating symptoms of CRC, improving the quality of life of patients and simultaneously prolonging their lifespan. Several studies recommend to include the DC-CIK therapy as adjuvant for currently applied anti-cancer therapy; however, the conclusions were drawn based on a small number clinical trials having possibly high heterogeneity and publication bias. Moreover, due to variability of studies (e.g., tumor stage, cell phenotype, cell purity) and parameters assessed throughout, the outcomes could not be unified. Despite promising outcomes obtained so far, there is no strong evidence that the DC-CIK therapy shows a clear advantage over currently used treatment options. Undoubtedly, combined immunotherapy and chemotherapy may have a synergistic effect on OS compared with chemotherapy alone. However, the definite efficacy of DC-CIK therapy is still not explicit and warrants evaluation.

## Author Contributions

PM and JF provided the overall concept and framework of the manuscript. AG, PM, and MZ researched and identified appropriate articles. PM and AG participated in writing the manuscript. PM, JF, and MZ revised the manuscript. All authors approved the final version of the manuscript.

## Conflict of Interest Statement

The authors declare that the research was conducted in the absence of any commercial or financial relationships that could be construed as a potential conflict of interest.

The reviewer VA and handling Editor declared their shared affiliation, and the handling Editor states that the process nevertheless met the standards of a fair and objective review.

## Abbreviations

ADCC: antibody-dependent cell-mediated cytotoxicity
APCs: antigen presenting cells
CIK: cytokine-induced killer cells
CRC: colorectal cancer
CTT: cytotoxic T lymphocytes
DCs: dendritic cells
DTH: Delayed Type Hypersensitivity
ER: endoplasmic reticulum
i-DCs: induced DCs
IDO: indoleamine 2,3-dioxygenase
IFN-γ: interferon gamma
IFNs-I: interferons type I
IL-1: interleukin-1
IL-2: interleukin-2
IL-6: interleukin-6
IL-12: interleukin-12
LAKs: lymphokine-activated killers
mDCs: myeloid dendritic cells
MHC: major histocompatibility complex
MST: medium survival time
NOD: nucleotide oligomerization domain
OS: overall survival
PBMS: peripheral blood mononuclear cells
pDCs: plasmacytoid dendritic cells
PFS: progression-free survival
TAAs: tumor associated antigens
TAP: transporters for antigen presentation
Th cell: T helper cell
